# Adverse events of active and placebo groups in SARS-CoV-2 vaccine randomized trials: A systematic review

**DOI:** 10.1016/j.lanepe.2021.100253

**Published:** 2021-10-28

**Authors:** Martina Amanzio, Dimos D Mitsikostas, Fabio Giovannelli, Massimo Bartoli, Giuseppina Elena Cipriani, Walter A Brown

**Affiliations:** a Department of Psychology, University of Turin, Via Verdi 10, 10124 Turin, Italy; b First Department of Neurology, Aeginition Hospital, National and Kapodistrian University of Athens, 72-74 V. Sofia's Avenue, Athens 11528, Hellas, Greece; c Section of Psychology - Department of Neuroscience, Psychology, Drug Research and Child's Health (NEUROFARBA), University of Florence, Via di San Salvi 12, 50135 Florence, Italy; d Department of Psychiatry and Human Behavior, Brown University, 69 Brown Street Providence, RI 02912, USA

**Keywords:** adverse events, nocebo effect, placebo, randomized control trials, SARS-CoV-2 vaccines

## Abstract

**Background:**

For safety assessment in clinical trials, adverse events (AEs) are reported for the drug under evaluation and compared with AEs in the placebo group. Little is known about the nature of the AEs associated with clinical trials of SARS-CoV-2 vaccines and the extent to which these can be traced to nocebo effects, where negative treatment-related expectations favor their occurrence.

**Methods:**

In our systematic review, we compared the rates of solicited AEs in the active and placebo groups of SARS-CoV-2 vaccines approved by the Western pharmaceutical regulatory agencies.

We implemented a search strategy to identify trial-III studies of SARS-CoV-2 vaccines through the PubMed database. We adopted the PRISMA Statement to perform the study selection and the data collection and identified three trial: two mRNA-based (38403 participants) and one adenovirus type (6736 participants).

**Findings:**

Relative risks showed that the occurrence of AEs reported in the vaccine groups was higher compared with the placebo groups. The most frequently AEs in both groups were fatigue, headache, local pain, as injection site reactions, and myalgia. In particular, for first doses in placebo recipients, fatigue was reported in 29% and 27% in BNT162b2 and mRNA-1273 groups, respectively, and in 21% of Ad26.COV2.S participants. Headache was reported in 27% in both mRNA groups and in 24% of Ad26.COV2.S recipients. Myalgia was reported in 10% and 14% in mRNA groups (BNT162b2 and mRNA-1273, respectively) and in 13% of Ad26.COV2.S participants. Local pain was reported in 12% and 17% in mRNA groups (BNT162b2 and mRNA-1273, respectively), and in 17% of Ad26.COV2.S recipients. These AEs are more common in the younger population and in the first dose of placebo recipients of the mRNA vaccines.

**Interpretation:**

Our results are in agreement with the expectancy theory of nocebo effects and suggest that the AEs associated with COVID-19 vaccines may be related to the nocebo effect.

**Funding:**

Fondazione CRT - Cassa di Risparmio di Torino, IT (grant number 66346, “GAIA-MENTE” 2019).

## Introduction

**Research in context**

**Evidence before this study**

Adverse events (AEs) of drugs are a central feature of safety assessment information. It is known that randomized clinical trials provide a perspective for understanding the role of negative expectations in the active drug group and the AEs of a placebo treatment described as a nocebo effect.

**Added value of this study**

Little is known about the nature of the AEs associated with clinical trials of SARS-CoV-2 vaccines and the extent to which these are nocebo effects, where negative treatment-related expectations favor their occurrence. To bridge this gap, our study aims to analyze the phase III safety data of vaccines approved to date with respect to the solicited AE profiles in the active and placebo groups. We found that AEs such as fatigue, headache, and pain (as local injection site reaction and myalgia) were the most commonly reported in both the active drug and the placebo arms, although in active vaccine arms they were higher.

The AEs of fatigue, headache, and pain are more common in the younger population and in the first dose of mRNA placebo recipients.

**Implication of all the available evidence**

Our results suggest that a substantial proportion of solicited AEs are not a result of the vaccine per se but are, in fact, nocebo effects. Awareness of the nocebo effect in placebo recipients of the vaccine trials may lead to a greater participation in the COVID-19 immunization and to a greater protection from infection.

Alt-text: Unlabelled box

Adverse events (AEs) of drugs are a central feature of the information that goes into clinical treatment decisions. It is known that by analyzing safety outcomes in placebo groups, randomized clinical trials (RCTs) provide a perspective for understanding the role of negative expectations in treatment outcomes – the nocebo effect, originally introduced to describe the negative effects of a placebo treatment.^1^

Different contextual and patients related factors leading to nocebo effects have been identified.^2^^,^^3^ Placebo groups from RCTs provide the data to analyze nocebo effects in terms of the occurrence of adverse reactions and treatment non-adherence in term of drop out. In a RCT, subjects know that they can receive either the active drug or the placebo and, accordingly, they are informed about the AEs they may experience. This can have a significant impact on the experience of associated discomfort. Indeed, drugs that produce more AEs cause highest symptomatic effects, even in the placebo groups,^4^, ^5^, ^6^, ^7^, ^8^ and consequently higher dropout rates due to a negative treatment outcome. Significantly, Benedetti et *al*.^9^ have recently demonstrated an involvement of hypothalamic-pituitary-adrenal activity and state anxiety in AEs reporting after placebo administration.

AEs may also be related to disease symptoms as reported for immunization with non-live vaccines. This highlights that symptoms are not always caused by the vaccine, but may be due to negative expectations and linked to nocebo risks.^10^ The reason why these AEs occur is unclear, and understanding the underlying mechanisms is an ongoing challenge.

However, to date, no study has assessed the AEs in the placebo control groups associated with COVID-19 vaccines. With this aim, we conducted an analysis of the solicited AEs in clinical trials for SARS-CoV-2 vaccines. We analyzed both active recipients and placebo groups, in order to test whether any of these AEs might be associated with nocebo effects.

## Methods

We implemented a search strategy to identify through the PubMed database (https://pubmed.ncbi.nlm.nih.gov), trial-III studies of SARS-CoV-2 vaccines published until 1 July 2021. No filter or limits were used. We adopted the "PRISMA Statement"^11^ to perform the study selection and the data collection (Supplementary Table 1).

We only considered trials approved by Western pharmaceutical regulatory agencies - i.e. the European Medicine Agency (EMA) or the Food and Drug Administration (FDA) - as safety data could cross-checked with results included in trial publications.

As inclusion criteria, we considered studies in which the placebo control group was treated with a saline solution and data collected considering the adult population (> 18 years).

Study selection was performed independently by three authors (M.A., M.B., G.E.C.), according to the pre-defined eligibility criteria. Cohen's K was used to assess the level of agreement in accordance with the inclusion and exclusion of the studies.

A total of three studies, out of 171, met the inclusion criteria and thus were included in our analysis (see the flow chart in Supplementary Figure 1). Risk of Bias of the three included studies was considered.^12^^,^^13^ Particularly, we analyzed studies concerning three different vaccines: BNT162b2,^14^ mRNA-1273,^15^ and Ad26.COV2.S.^16^ We identified ten clinical trials: five were clinical trials with mRNA-based vaccines (BNT162b2: no. two; mRNA-1273: no. three), and five were clinical trials using an adenovirus vector vaccine (Ad26.COV2.S).

The trials performed with AZD1222,^14^ approved only by the EMA but not by the FDA, were excluded as the control groups were treated with meningococcal vaccine for serogroups A, C, W, and Y (MenACWY), or with MenACWY (as first dose) and saline (as second dose). Only one trial (COV005) for AZD1222 had administered saline in the placebo group; however, this trial was not included in the interim primary efficacy analyses.

With regard to data abstraction, each of the three selected studies was coded using a structured coding scheme, including information on report identification, methodology, subjects, and treatment.

During the validity assessment phase, two different authors (M.B. and G.E.C.) performed the assessment of methodological quality independently. Disagreements that occurred in this phase were solved through discussion between all the authors.

Cohen's K used to assess the level of agreement in accordance with the inclusion and exclusion of the studies: % agreement 100; Cohen's K: 1.

The Cochrane Collaboration's tool for assessing risk of bias^12^^,^^13^ was used in order to consider: [a] selection bias (concerning ‘sequence generation’ and ‘allocation concealment’); [b] performance bias (‘blinding of participants and personnel’); [c] detection bias (‘blinding of outcome assessment’); [d] attrition bias (‘incomplete outcome data’); [e] reporting bias (‘selective outcome reporting’). Particularly, we assessed separately the domains “incomplete outcome data” and “selective outcome reporting” for subjects withdrawing due to AEs and occurrence of AEs (including serious AEs).

Data from the selected trials were extracted by three authors (M.A., M.B., G.E.C.).

As ascertainment strategy based on the solicited safety set, for all studies, the AEs were collected in both vaccine and placebo group arms. For RCTs involving mRNA technology vaccines, AEs data from both the first and the second dose were also extracted; whereas, for RCTs involving the viral vector type vaccine, only AEs data from the first and unique dose were considered. Moreover, we also collected the unsolicited adverse reactions (reported within 28 days after the injection) in terms of severe symptoms (Serious Adverse Events, SAEs), and deaths in the three selected trials.

Finally**,** variables such as sample size, gender, age, withdrawals, diagnosis of COVID-19 and ethnicity, were also collected from the full analysis sets.

In the selected trials, the AEs were coded according to the Medical Dictionary for Regulatory Activities (MedDRA). In each trial, AEs data were reported as the total number of participants in a specific group, the number and the percentage of participants with specified adverse reaction.

Solicited AEs included in the selected trials refer to – a list of events and symptoms that participants were specifically asked to record using electronic diaries – within seven days of inoculation. Solicited AEs were extracted from the safety set of each study. We only considered and included in the database AEs that were present in at least two out of three trials of each vaccine. We identified eleven categories of solicited AEs, both systemic symptoms and injection site reactions, reported in the studies. In particular, we grouped the terms arthralgia and joint pain, myalgia and muscle pain, nausea and vomiting, local erythema and redness, according to MedDRA classification (Table 1).

**Table 1:** Solicited adverse events categories (n = 11) observed in the vaccine and the placebo groups (reported in at least two out of the three included studies) divided into systemic reactions and local injection site reactions.

	BNT162b2	mRNA-1273	AD26.COV2.S
**Systemic reactions**			
Arthralgia / Joint Pain	Joint pain	Arthralgia	
Chills	Chills	Chills	
Fatigue	Fatigue	Fatigue	Fatigue
Fever	Fever	Fever	Fever
Headache	Headache	Headache	Headache
Myalgia / Muscle pain	Muscle pain	Myalgia	Myalgia
Nausea / Vomiting	Vomiting	Nausea / Vomiting	Nausea
Antipyretic /Analgesic use b	Use of antipyretic or pain medication		Antipyretic/Analgesic Use
**Local injection site reactions**			
Local Erythema / Redness	Redness	Erythema (redness)	Erythema
Local Pain	Pain	Pain	Pain
Local Swelling	Swelling	Swelling	Swelling

^a^ The categorization into eleven categories follows the classification of solicited AEs

b The antipyretic/analgesic use is a systemic reaction effect considered as an AE in the selected trials

Percentages of patients reporting the solicited AEs, with 95% confidence intervals (CIs), have been expressed for all reported outcome measures. Non-overlapping CIs of the percentages of AEs indicate significant differences among the different categories.

For mRNA vaccines (BNT162b2 and mRNA-1273), percentages with 95% CIs were calculated for both the first and the second dose. Moreover, safety data were also analyzed by taking into account two different age ranges, as reported in the original trials: (a) BNT162b2: 18-54 years and 55 years and older; (b) mRNA-1273: 18-64 years and 65 years and older; (c) Ad26.COV2.S (single dose): 18-59 years and 60 years and older.

For each study, relative risk (RR) of solicited AEs was calculated in order to compare vaccine and placebo groups for all reported symptoms.

The funding source has no roles in study design, data collection, data analysis, interpretation, and writing of the report.

## Results

The risk of bias was generally low in all trials. However, one study (of BNT162b2 vaccine) presented an unclear risk concerning performance bias (blinding of participants and personnel), while an unclear risk was found with respect to detection bias (blinding of outcome assessment) in another study (Ad26.COV2.S vaccine).

With regard to the characteristics of participants included in the full analysis set and divided into vaccine and placebo groups, the mean age and proportion of male/female subjects appears comparable among the three studies in both vaccine and placebo groups. It is noteworthy that the ethnicity most represented in all three studies is Caucasian (Supplementary Tables 2 and 3).

The total number of subjects included in the safety population set for BNT162b2 was 8080 (4040 in both the vaccine and placebo groups), for mRNA-1273 was 30323 (15168 in the vaccine group and 15155 in the placebo) and for Ad26.COV2.S was 6736 (3356 in the vaccine group and 3380 in the placebo group). The number and percentage (95 % CI) of AEs across the different groups are reported in tables 2 and 3 considering active and placebo recipients, respectively. The percentage of AEs for different age groups of all trials are reported in the Supplementary Table 4.

**Table 2:** Safety set of solicited adverse events – any grade - reported in the active groups for mRNA-1273 for BNT162b2, mRNA-1273, and AD26.COV2.S.

	BNT162b2				mRNA-1273				AD26.COV2.S	
	1^st^ dose (N = 4040)		2^nd^ dose (N = 3705)		1^st^ dose (N = 15168)		2^nd^ dose (N = 14677)		1^st^ single-dose (N = 3356)	
AEs	nAEs (%)	95% CI	nAEs (%)	95% CI	nAEs (%)	95% CI	nAEs (%)	95% CI	nAEs (%)	95% CI
Arthralgia/joint pain	406 (10·0)	9·1 – 11·0	772 (20·8)	19·5 – 22·1	2511 (16·6)	16·0 – 17·1	6284 (42·8)	42·0 – 43·6		
Antipyretic/Analgesic use	996 (24·7)	23·3 – 26·0	1570 (42·4)	42·4 – 44·0					668 (19·9)	18·6 – 21·3
Any local AR					12765 (84·2)	83·6 – 84·7	13006 (88·6)	88·1 – 89·1	1685 (50·2)	48·5 – 51·9
Any systemic AEs					8320 (54·9)	54·1 – 55·6	11652 (79·4)	78·7 – 80·0	1850 (55·1)	53·4 – 56·8
Chills	434 (10·7)	9·8 – 11·7	1114 (30·1)	28·6 – 31·5	1253 (8·3)	7·8 – 8·7	6482 (44·2)	43·4 – 45·0		
**Fatigue**	**1700 (42·1)**	**40·6 – 43·6**	**2086 (56·3)**	**54·7 – 57·9**	**5635 (37·2)**	**36·4 – 37·9**	**9582 (65·3)**	**64·5 – 66·1**	**1283 (38·2)**	**36·6 – 39·9**
Fever	111 (2·7)	2·2 – 3·3	512 (13·8)	12·7 – 14·9	115 (0·8)	0·6 – 0·9	2278 (15·5)	14·9 – 16·1	302 (9·0)	8·0 – 10·0
**Headache**	**1413 (35·0)**	**33·5 – 36·4**	**1732 (46·7)**	**45·1 – 48·4**	**4951 (32·6)**	**31·9 – 33·4**	**8602 (58·6)**	**57·8 – 59·4**	**1306 (38·9)**	**37·3 – 40·6**
Local erythema/redness	189 (4·7)	4·0 – 5·3	243 (6·6)	5·8 – 7·4	430 (2·8)	2·6 – 3·1	1257 (8·6)	8·1 – 9·0	245 (7·3)	6·4 – 8·2
**Local pain**	**3186 (78·9)**	**77·6 – 80·1**	**2730 (73·7)**	**72·3 – 75·1**	**12690 (83·7)**	**83·1 – 84·3**	**12943 (88·2)**	**87·7 – 88·7**	**1632 (48·6)**	**46·9 – 50·3**
Local swelling	250 (6·2)	5·4 – 6·9	256 (6·9)	6·1 – 7·7	932 (6·1)	5·8 – 6·5	1789 (12·2)	11·7 – 12·7	178 (5·3)	4.5 – 6·1
**Myalgia/muscle pain**	**738 (18·3)**	**17·1 – 19·5**	**1260 (34·0)**	**32·5 – 35·5**	**3441 (22·7)**	**0·22 – 23·4**	**8508 (58·0)**	**57·2 – 58·8**	**1113 (33.2)**	**31·6 – 34·8**
Nausea/vomiting	37 (0·9)	0·6 – 1·2	51 (1·4)	1·0 – 1·8	1262 (8·3)	7·9 – 8·8	2785 (19·0)	18·3 – 19·6	477 (14·2)	13 – 15·4

Abbreviations: N= sample size. AEs= Adverse Events. n AEs= number of Adverse Events. CI= Confidence Interval. AR= Adverse reaction.

a 95% confidence intervals are indicated (inferior value of the interval - superior value of the interval).

b The bold lines represent the most frequently reported symptoms.

c The absence of an AE in one trial, which was reported in the others, was considered in terms of an absence of the symptom and depicted as grey cells.

**Table 3:** Safety set of solicited adverse events - any grade - reported in the placebo groups for mRNA-1273 for BNT162b2, mRNA-1273, and AD26.COV2.S.

	BNT162b2				mRNA-1273				AD26.COV2.S	
	1st dose (N = 4040)		2nd dose (N = 3699)		1st dose (N = 15155)		2nd dose (N = 14566)		1st single-dose (N = 3380)	
AEs	nAEs (%)	95% CI	nAEs (%)	95% CI	nAEs (%)	95% CI	nAEs (%)	95% CI	nAEs (%)	95% CI
Arthralgia/joint pain	247 (6·1)	5·4 – 6·9	170 (4·6)	3·9 – 5·3	1783 (11·8)	11·3 – 12·3	1569 (10·8)	10·3 – 11·3		
Antipyretic/Analgesic use	545 (13·5)	12·4 – 14·5	427 (11·5)	10·5 – 12·6					191 (5·7)	4·9 – 6·4
Any local AR					2997 (19·8)	19·1 – 20·4	2735 (18·8)	18·1 – 19·4	657 (19·4)	18·1 – 20·8
Any systemic AEs					6399 (42·2)	41·4 – 43·0	5323 (36·5)	35·8 – 37·3	1185 (35·1)	33·5 – 36·7
Chills	203 (5·0)	4·4 – 5·7	125 (3·4)	2·8 – 4·0	878 (5·8)	5·4 – 6·2	809 (5·6)	5·2 – 5·9		
**Fatigue**	**1172 (29·0)**	**27·6 – 30·4**	**756 (20·4)**	**19·1 – 21·7**	**4133 (27·3)**	**26·6 – 28·0**	**3403 (23·4)**	**22·7 – 24·0**	**728 (21·5)**	**20·2 – 22·9**
Fever	27 (0·7)	0·4 – 0·9	14 (0·4)	0·2 – 0·6	44 (0·3)	0·2 – 0·4	43 (0·3)	0·2 – 0·4	20 (0·6)	0·3 – 0·9
**Headache**	**1100 (27·2)**	**25·9 – 28·6**	**735 (19·9)**	**18·6 – 21·2**	**4027 (26·6)**	**25·9 – 27·3**	**3410 (23·4)**	**22·7 – 24·1**	**802 (23·7)**	**22·3 – 25·2**
Local erythema/redness	45 (1·1)	0·8 – 1·4	26 (0·7)	0·4 – 1·0	67 (0·4)	0·3 – 0·5	56 (0·4)	0·3 – 0·5	131 (3·9)	3·2 – 4·5
**Local pain**	**488 (12·1)**	**11·1 – 13·1**	**372 (10·1)**	**9·1 – 11·0**	**2658 (17·5)**	**16·9 – 18·1**	**2477 (17·0)**	**16·4 – 17·6**	**564 (16·7)**	**15·4 – 17·9**
Local swelling	32 (0·8)	0·5 – 1·1	16 (0·4)	0·2 – 0·6	52 (0·3)	0·3 – 0·4	49 (0·3)	0·2 – 0·4	53 (1·6)	1·1 – 2·0
**Myalgia/muscle pain**	**398 (9·9)**	**8·9 – 10·8**	**260 (7·0)**	**6·2 – 7·9**	**2071 (13·7)**	**13·1 – 14·2**	**1809 (12·4)**	**11·9 – 13·0**	**430 (12·7)**	**11·6 – 13·8**
Nausea/vomiting	37 (0·9)	0·6 – 1·2	30 (0·8)	0·5 – 1·1	1074 (7·1)	6·7 – 7·5	934 (6·4)	6·0 – 6·8	327 (9·7)	8·7 – 10·7

Abbreviations: N= sample size. AEs= Adverse Events. n AEs= number of Adverse Events. CI= Confidence Interval. AR= Adverse Reaction.

a 95% confidence intervals are indicated (inferior value of the interval - superior value of the interval).

b The bold lines represent both the most frequently reported symptoms and a decrease in reported adverse reactions following the second dose in comparison to the first dose (only the difference between mRNA-1273 groups for local pain was not statistically significant).

c The absence of an AE in one trial, which was reported in the others, was considered in terms of an absence of the symptom and depicted as grey cells.

RRs showed that, in each study, the probability of occurrence of almost all AEs reported in the vaccine groups was higher compared to the placebo groups (Table 4).

**Table 4:** Relative risk (95 % CI) of solicited adverse events – any grade - for BNT162b2, mRNA-1273, and Ad26.COV2.S (safety set).

AEs	Vaccine	Active group		Placebo group		RR	lower CI	upper CI
		N	n_AEs (%)	N	n_AEs (%)			
Arthralgia/joint pain	BNT162b2	4040	406 (10·05)	4040	247 (6·11)	1·644	1·412	1·914
	mRNA-1273	15168	2511 (16·55)	15155	1783 (11·76)	1·407	1·330	1·489
	Ad26.COV2.S					–	–	–
Antipyretic/analgesic use	BNT162b2	4040	996 (24·65)	4040	545 (13·49)	1·828	1·662	2·009
	mRNA-1273					–	–	–
	Ad26.COV2.S	3356	668 (19·90)	3380	191 (5·65)	3·522	3·021	4·107
Any local AR	BNT162b2					–	–	–
	mRNA-1273	15168	12765 (84·15)	15155	2997 (19·77)	4·256	4·118	4·398
	Ad26.COV2.S	3356	1685 (50·20)	3380	657 (19·43)	2·583	2·393	2·788
Any systemic AEs	BNT162b2					–	–	–
	mRNA-1273	15168	8320 (54·85)	15155	6399 (42·22)	1·299	1·269	1·330
	Ad26.COV2.S	3356	1850 (55·12)	3380	1185 (35·06)	1·572	1·488	1·661
Chills	BNT162b2	4040	434 (10·74)	4040	203 (5·02)	2·138	1·820	2·511
	mRNA-1273	15168	1253 (8·26)	15155	878 (5·79)	1·426	1·312	1·550
	Ad26.COV2.S					–	–	–
**Fatigue**	**BNT162b2**	**4040**	**1700 (42·08)**	**4040**	**1172 (29·01)**	**1·451**	**1·366**	**1·541**
	**mRNA-1273**	**15168**	**5635 (37·15)**	**15155**	**4133 (27·27)**	**1·362**	**1·318**	**1·408**
	**Ad26.COV2.S**	**3356**	**1283 (38·23)**	**3380**	**728 (21·54)**	**1·775**	**1·643**	**1·918**
Fever	BNT162b2	4040	111 (2·74)	4040	27 (0·67)	4·111	2·706	6·246
	mRNA-1273	15168	115 (0·75)	15155	44 (0·29)	2·611	1·846	3·694
	Ad26.COV2.S	3356	302 (8·99)	3380	20 (0·59)	15·208	9·697	23·851
**Headache**	**BNT162b2**	**4040**	**1413 (34·97)**	**4040**	**1100 (27·23)**	**1·285**	**1·203**	**1·372**
	**mRNA-1273**	**15168**	**4951 (32·64)**	**15155**	**4027 (26·57)**	**1·228**	**1·186**	**1·272**
	**Ad26.COV2.S**	**3356**	**1306 (38·91)**	**3380**	**802 (23·73)**	**1·640**	**1·523**	**1·766**
Local erythema/redness	BNT162b2	4040	189 (4·67)	4040	45 (1·11)	4·200	3·043	5·796
	mRNA-1273	15168	430 (2·83)	15155	67 (0·44)	6·412	4·962	8·287
	Ad26.COV2.S	3356	245 (7·30)	3380	131 (3·87)	1·884	1·532	2·316
**Local pain**	**BNT162b2**	**4040**	**3186 (78·86)**	**4040**	**488 (12·08)**	**6·529**	**5·998**	**7·106**
	**mRNA-1273**	**15168**	**12690 (83·66)**	**15155**	**2658 (17·54)**	**4·770**	**4·605**	**4·941**
	**Ad26.COV2.S**	**3356**	**1632 (48·63)**	**3380**	**564 (16·68)**	**2·914**	**2·682**	**3·166**
Local swelling	BNT162b2	4040	250 (6·19)	4040	32 (0·79)	7·812	5·421	11·258
	mRNA-1273	15168	932 (6·14)	15155	52 (0·34)	17·908	13·556	23·656
	Ad26.COV2.S	3356	178 (5·30)	3380	53 (1·56)	3·383	2·498	4·579
**Myalgia/muscle pain**	**BNT162b2**	**4040**	**738 (18·26)**	**4040**	**398 (9·85)**	**1·854**	**1·655**	**2·078**
	**mRNA-1273**	**15168**	**3441 (22·68)**	**15155**	**2071 (13·66)**	**1·660**	**1·580**	**1·745**
	**Ad26.COV2.S**	**3356**	**1113 (33·16)**	**3380**	**430 (12·72)**	**2·607**	**2·358**	**2·883**
Nausea/vomiting	BNT162b2	4040	37 (0·91)	4040	37 (0·91)	1·000	0·635	1·574
	mRNA-1273	15168	1262 (8·32)	15155	1074 (7·08)	1·174	1·086	1·270
	Ad26.COV2.S	3356	477 (14·21)	3380	327 (9·67)	1·469	1·287	1·677

Abbreviations: AEs= Adverse Events. N= sample size. n AEs= number of Adverse Events. RR=Relative Risk. CI= Confidence Interval. AR= Adverse Reaction.

a The bold lines represent the most frequently reported symptoms.

The most frequently solicited AEs in the active and placebo groups were fatigue, headache, local pain as injection site reactions, and myalgia/muscle pain.

In particular, considering the first doses, fatigue was reported by 42% and 37% subjects after the first dose of BNT162b2 and mRNA-1273 respectively, and by 38% of the single dose of Ad26.COV2.S. The percentage of subjects reporting fatigue in the placebo group was 29% and 27% for the first dose of BNT162b2 and mRNA-1273 trials respectively, and 21% for the single dose of Ad26.COV2.S trial.

After the first dose, headache was reported by 35% of the BNT162b2 vaccine group and by 33% of the mRNA-1273 vaccine group. For the unique dose of Ad26.COV2.S vaccine, 39% of the subjects experienced headache. About 27% of placebo groups of BNT162b2 and mRNA-1273 reported headache after the first dose, while 24% of the subjects of the placebo group combined with Ad26.COV2.S vaccine exhibited such AE after the administration of the single dose.

As regards injection side reactions, local pain was reported after the first dose by 79% and 84% of the BNT62b2 and mRNA-1273 vaccine respectively, and by 49% of Ad26.COV2.S vaccine group after the single dose. Local pain was reported after the first dose by 12% and 17% of the subjects of the placebo groups combined with BNT162b2 and mRNA-1273 vaccine groups respectively, and by 17% of the placebo group combined with Ad26.COV2.S vaccine after the administration of the single dose.

Myalgia/muscle pain was reported by 18% of BNT162b2 vaccine group subjects and 23% of mRNA-1273 vaccine group participants after the first dose, and by 33% of subjects who received a single dose of Ad26.COV2.S. In the placebo groups, myalgia/muscle pain was reported by 10% and 14% of the BNT162b2 and mRNA-1273 trials respectively, after the first dose, and by 13% of Ad26.COV2.S after the single-dose.

**Fig. 1:**
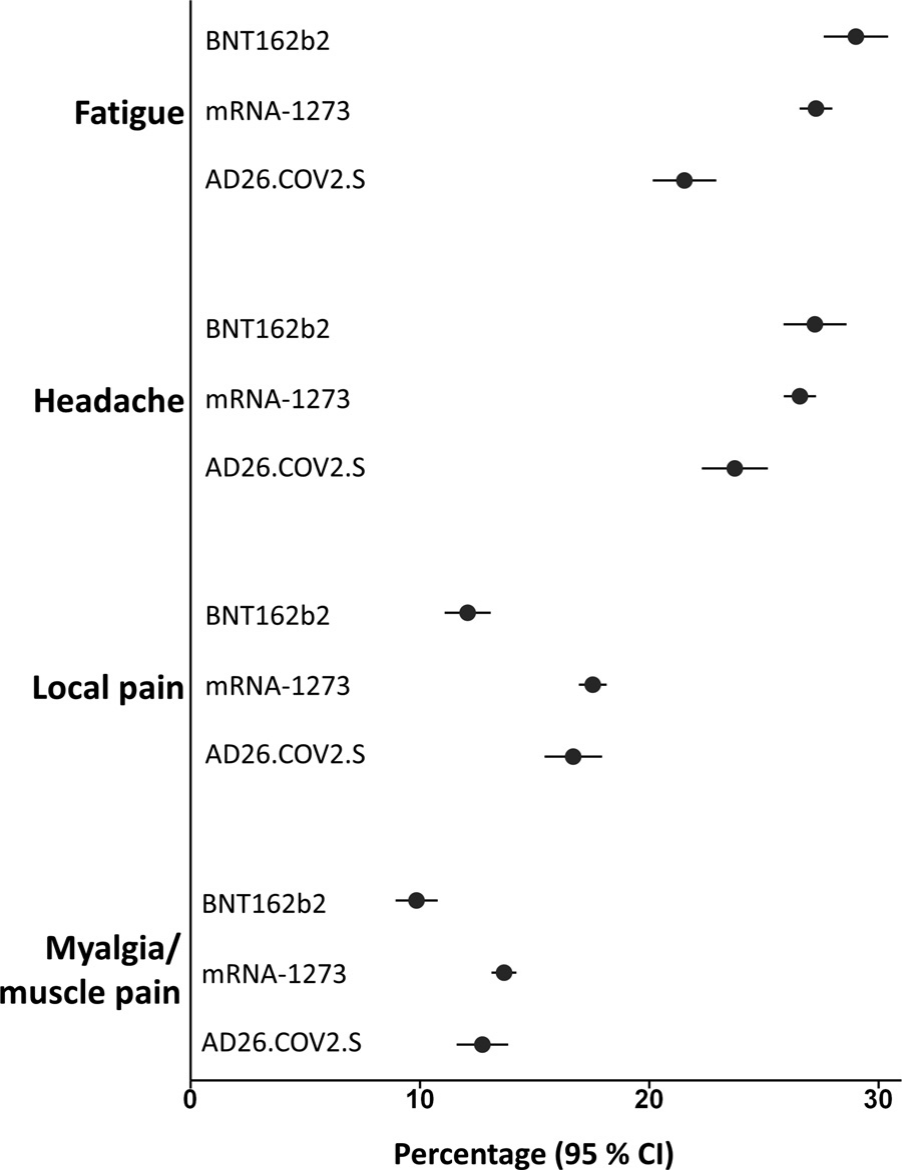
Percentage of fatigue, headache, local pain, and myalgia/muscle pain in the placebo groups. Percentage (95% CI) of solicited AEs in the placebo groups of mRNA based vaccines (first doses) and viral vector vaccine considering fatigue, headache, local pain, and myalgia/muscle pain.

The most frequently reported adverse reaction in the active groups was local pain, with a higher percentage frequency observed for the mRNA vaccines (BNT162b2: 79%; mRNA-1273: 84%; Ad26.COV2.S: 49%); whereas fatigue (BNT162b2: 29%; mRNA-1273: 27%; Ad26.COV2.S: 21%) and headache (BNT162b2: 27%; mRNA-1273: 27%; Ad26.COV2.S: 24%) were the most common AEs among placebo recipients (Fig. 1).

**Fig. 2:**
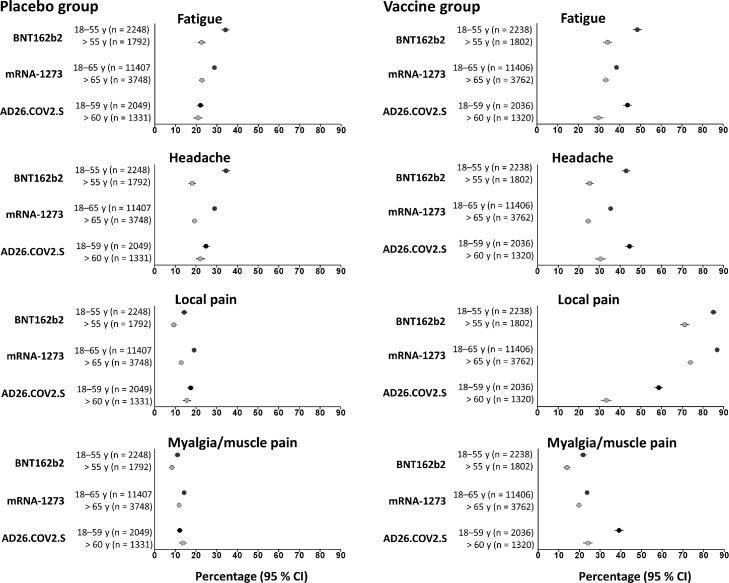
Percentage of fatigue, headache, local pain, and myalgia/muscle in younger and older placebo recipients, compared to the vaccine arms. Percentage (95% CI) of solicited AEs in younger and older placebo recipients, compared to the vaccine arms, of mRNA based vaccines (first doses) and viral vector vaccine considering fatigue, headache, local pain, and myalgia/muscle pain.

After the first dose, a significant increase of fatigue and headache was observed in younger subjects in the placebo groups of the mRNA vaccines (CI: 0·3 to 0·4) in comparison to the older groups. In contrast, the percentage of these adverse events (fatigue and headache) in the younger and older recipients was similar for placebo group of the viral vector type vaccine. Considering participants aged 18-54 years, fatigue and headache were reported more in the placebo groups combined with BNT162b2. Interestingly, in the vaccine recipients local pain was the most reported symptom, as injection site reaction, with a higher representation in the younger subjects (for the mRNA vaccines with a C.I. between 0·7-0·9, and for the viral vector type vaccine between 0·3-0·6). However, in the placebo recipients, this specific AE was reported in the same C.I., between 0·1-0·2, in the two age groups and for all three vaccines. The systemic side effect of myalgia/muscle pain was reported in vaccine recipients in a C.I. between 0·1 and 0·4, with a higher representation for younger subjects in the three groups, and considering placebo recipients between 0·1-0·2 for the two age groups (Fig. 2).

Finally, as shown in table 3, the percentage (95% CI) with AEs was less after the second dose of placebo. Specifically, among the systemic reactions, fatigue, headache, and myalgia/muscle pain were observed to a lesser extent after the second doses of placebo in comparison to the first doses. Particularly, fatigue decreased from 29% to 20% in the BNT162b2-associated group, and from 27% to 23% in mRNA-1273-associated group. Headache decreased from 27% to 20% in the BNT162b2-associated group and from 27% to 23% in mRNA-1273-associated group. Finally, myalgia/muscle pain decreased from 10% to 7% in the BNT162b2-associated group and from 14% to 12% in mRNA-1273-associated group.

On the contrary, considering local pain as a specific adverse reaction, we observed a decrease following the second dose of placebo for the BNT162b2-associated group (from 12% to 10%) and not for the mRNA-1273-associated group.

Unsolicited AEs were more common in the vaccine groups than in the placebo groups (BNT162b2: 27% vs 12%; mRNA-1273: 22% vs 19%; Ad26.COV2.S: 13% vs 12%). SAEs observed in the active and placebo groups of the three different vaccines occurred at a similar rate. Particularly, any type of SAEs was reported in less than 1% of both vaccine and placebo groups for all the trials (BNT162b2: 0·6% vs 0·5%; mRNA-1273: 0·5% vs 0·6%; Ad26.COV2.S: 0.4% in both vaccine and placebo groups). Interestingly, SAEs considered related by the investigators to the administration of the drug or placebo were < 0·1% for all the studies analyzed. Furthermore, deaths only occurred in < 0·1% in both vaccine and placebo groups (Supplementary Table 5).

## Discussion

The aim of our study was to examine the phase III of COVID-19 vaccines approved to date. To this end, we analyzed the solicited AE profiles in the active and placebo groups, as recorded in the reactogenicity subsets.

In this review, we have shown, for the first time in the literature, that inducing specific expectations in placebo recipients can provoke AEs in terms of systemic and local symptoms.

We found that the solicited AEs profile in the placebo arms of the studies is comparable to those of the vaccine with which placebo was compared, although the percentage was higher with vaccine (as shown in table 4). Specifically, some adverse reactions, such as fatigue, headache, and pain (i.e., injection site reaction and myalgia), which were the most common AEs in the active groups, were also observed in the placebo groups, with a higher C.I. for fatigue and headache reported in placebo recipients.

In particular, for the first doses in placebo recipients, the most reported AEs were fatigue and headache, while the most reported AEs in the vaccine group was local pain.

Among the systemic reactions, fatigue, headache, and myalgia/muscle pain were observed to a lesser extent after the second doses of placebo, in comparison to the first doses. Conversely, in the vaccine groups, we observed an increase of almost all AEs following the second dose, with the exception of local pain.

Interestingly, younger participants showed higher reporting of fatigue and headache both in the placebo groups of the mRNA vaccines and after the first doses. Considering the total subset of participants, systemic reactions of fatigue, headache, and myalgia decreased substantially from the first to the second dose of placebo combined with the mRNA vaccines (with the exception of local pain for mRNA-1273, as injection site reaction). Conversely, in the active groups, the same symptoms increased substantially after the second doses (with the exception of local pain for BNT162b2, as injection site reaction).

We have considered AEs categorized in the ascertainment strategy as structured recording, where symptoms were collected using a checklist or diary cards. In particular, solicited AEs were recorded using electronic diaries, through the assessment of systematic reactions and local injection site symptoms. The recording of solicited AEs, assessed in the placebo recipients after the first doses, and included in a checklist, instead of spontaneous reports or unsolicited symptoms, allowed us to analyze the role of negative expectations in treatment outcomes. In addition, the results of the second doses of vaccines and placebos allowed us to investigate aspects not only related to the negative expectations associated with a new treatment, but also in terms of learning from previous experience, as a conditioning phenomenon.

Non-pharmacodynamic factors, such as expectation alone, analyzed in the placebo groups of the considered trials after the first treatment dose, may have triggered distressing symptoms—as an effect of the nocebo phenomenon.^1^^,^^17^ Self-fulfilling prophecy is a phenomenon whereby the belief that a future event will occur contributes to the actual occurrence of that adverse reaction. It plays a crucial role in modelling experiences and can be considered causal, rather than simply predictive.^18^^,^^19^ Such beliefs, as response expectations, can influence health outcomes,^19^^,^^20^ as previously seen in allergology, in the treatment of muscle tension, for gastrointestinal disorders, and erectile dysfunction, in asthmatic patients and during generic substitution.^21^, ^22^, ^23^, ^24^, ^25^, ^26^, ^27^, ^28^, ^29^ This phenomenon may be particularly relevant during the current state of pandemic emergency and when testing new vaccines.^30^^,^^31^ Furthermore, it has been observed that the reporting of AEs was more common in patients with negative expectations when taking a new drug.^32^ Fear and anxiety of experiencing adverse reactions due to the treatment may be increased. These observations are in line with what has been previously reported with respect to non-live vaccines, as symptoms occurring after immunization are not always caused by the vaccine itself, but may be due to negative expectation and linked to nocebo risks.^10^ These concerns may have an impact on the proportion of AEs reported in RCTs.In several studies of rituximab administration for rheumatoid arthritis, the proportion of AEs decreased by 50% with subsequent doses, and this result, (without pharmacological explanation), could be related to decreased anxiety in participants.^33^, ^34^, ^35^ This is in line with what we observed for systemic reactions, such as fatigue and headache in placebo recipients of the new mRNA vaccines. These two systemic reactions were the most represented solicited symptoms in placebo recipients and decreased after the second dose (with a decrease also observed for myalgia). Notably, these symptoms often occur in healthy people who do not take medication.^36^ In their study, considering different clinical conditions, Howick and colleagues^37^ found that headache, fatigue and myalgia were among the most frequently reported systemic AEs in the placebo groups of the analyzed RCTs. It is interesting to note that in the vaccine recipients at the first doses, the most reported AE was local pain, while in the placebo recipients the more represented AEs were systemic reactions, such as fatigue and headache. Moreover, considering the second doses*,* the systemic AEs decreased in placebo recipients, while all the solicited AEs increased in the vaccine groups. Such aspect may be influenced by several factors, including conditioning. Significantly, our results seem to suggest a role for nocebo in systemic adverse reactions, as mild symptoms, most of which are not vaccine related.

Data from our study support the hypothesis that a substantial proportion of the AEs of COVID-19 vaccines may be related to nocebo effects. The fairly high proportions of placebo and vaccine recipients who have experienced AEs, and the adverse reactions observed, may suggest prevention strategies to promote a possible greater adherence to the vaccination campaign. The media and health professionals could potentially reduce these side effects through positive framing and by raising awareness of the nocebo effect. Unfortunately, the presence of the current infodemic risk,^38^ in which negative information related to clinical trials of COVID-19 vaccines, may lead to an escalation in the occurrence of AEs.^30^^,^^31^^,^^39^ The Internet as a source of medical information could lead to limited trust in new treatments. Indeed, negative publicity of statin-induced myalgia has led to high rates of statin discontinuation, use of second-line agents, and increased rates of cardiovascular events among participants of an RCT of the statin atorvastatin in the non-blind extension phase.^40^ On the other hand, and more recommended as a way of proceeding, as it was suggested by Barnes et *al*.,^41^ the empirical studies that analyzed positive framing emphasized how it is a promising strategy to reduce nocebo effects. However, they also revealed some important open questions, including the best method of communicating AEs, the optimal statistical presentation, whether framing affects the perceived absolute risk of side effects, and what psychological mechanisms underlie framing effects. The authors pointed out that future research will be vital in understanding the circumstances in which framing is most likely to be effective.^41^ In particular, awareness of the nocebo effect may lead to a greater participation in the COVID-19 immunization and to a greater protection from infection.^29^ Significantly, pointing out that the anticipation or fear of an adverse reaction can become a self-fulfilling prophecy (by causing the misattribution of unrelated, pre-existing symptoms to the drug being evaluated) may in itself help to obviate the presentation of adverse reactions. It may also be helpful to discuss the nocebo phenomenon explicitly at the time of trial recruitment and to explain how somatic symptoms caused by pre-existing medical illnesses, or by anxiety, can be misattributed to an active medication. In particular, strategies to minimize nocebo-related risks and to improve adherence to vaccine inoculation should be considered and discussed to possibly modify negative expectations associated with a new treatment. Positive framing has previously been shown to be an effective strategy to prevent AEs in the influenza vaccination campaign.^42^^,^^43^ It would be useful to focus on benefits rather than negative effects, e.g. by highlighting the proportion of patients who usually tolerate the treatment well, without experiencing particular side effects. Significantly, it would be also crucial to emphasize how the levels of occurrence of SAEs in the analyzed trials were similar in vaccine and placebo recipients, and how they were defined by the authors as unrelated to vaccination and in line with the expected background rate in the general population.^14^, ^15^, ^16^

Our study leads to the important hypothesis that phase III trials of COVID-19 vaccines could cause the nocebo effect and alter their safety outcomes. The emergency authorization of SARS-CoV-2 vaccines also may lead the population to fear and anxiety about the safety of these new drugs, triggering negative expectations associated with immunization.

## Limitations of the study

The limitation of this study is that the data are taken from published articles with information provided in a summary format and individual patient data were not available. In addition, the data obtained from the studies represent a further criticality due to the short duration of the phase III safety follow-up. The evaluation of long-term safety studies cannot be done in the context of maintaining a placebo group for the planned follow-up period of two years after the second dose. This makes the generalization of the results to the general population more uncertain. Furthermore, as we considered 10 clinical trials in three selected studies, we could not perform a meta-analysis. Future studies should consider and deeply explore the role played by nocebo effects in order to improve patients’ adherence to these lifesaving therapies.

## Contributors

MA designed the study. MA and FG were responsible for acquisition, analysis, and interpretation of data. All authors wrote the draft of the manuscript. MA, FG, WB, and DM participated in the critical revision of the manuscript for important intellectual content. MA, FG, MB, and GEC selected the articles and extracted the data. MA was responsible for funding acquisition. MA, WB, and DM supervised the entire project.

## Declaration of Competing Interest

All authors declare no conflict of interest.

## Appendix Supplementary materials

Image, application 1

## Footnotes

Supplementary material associated with this article can be found in the online version at doi:10.1016/j.lanepe.2021.100253.
